# Supplementary material for: Relating simulation studies by provenance—Developing a family of Wnt signaling models
Source: PLoS Comput Biol. 2021 Aug 5;17(8):e1009227. doi: 10.1371/journal.pcbi.1009227 (PMC8407594; doi:10.1371/journal.pcbi.1009227)
Supplement: S1 Appendix — We show the references to additional studies that contain entities used by some of the 19 Wnt simulation studies. (PDF) [file pcbi.1009227.s001.pdf]

Supporting Information: Relating simulation studies by provenance — Developing a family of Wnt signaling models

Kai Budde (kai.budde@uni-rostock.de)

July 9, 2021

1 List of names and references of studies shown in provenance graphs

This list shows the names used in the provenance graphs as well as the corresponding references.

Wnt simulation studies:

- Chen et al. 2014 [1]
- Cho et al. 2006 [2]
- Goldbeter and Pourquié 2008 [3]
- Haack et al. 2015 [4]
- Haack et al. 2020 [5]
- Kim et al. 2007 [6]
- Kogan et al. 2012 [7]
- Krüger and Heinrich 2004 [8]
- Lee et al. 2001 [9]
- Mazemondet et al. 2012 [10]
- Mirams et al. 2010 [11]
- Padala et al. 2017 [12]
- Rodriguez et al. 2007 [13]
- Sick et al. 2006 [14]
- Staehlke et al. 2020 [15]
- van Leeuwen et al. 2007 [16]
- van Leeuwen et al. 2009 [17]
- Wang et al. 2013 [18]
- Wawra et al. 2007 [19]

Additionally used studies:

- Bafico et al. 2001 [20]
- Bernard et al. 2006 [21]
- Bourhis et al. 2010 [22]
- Brown et al. 2004 [23]
- Cho et al. 2003 [24]
- Dajani et al. 2003 [25]
- Dequeant et al. 2006 [26]
- Galli et al. 2006 [27]
- Goh and Sorkin 2013 [28]
- Hannoush 2008 [29]
- Hernandez et al. 2012 [30]
- Hirata et al. 2004 [31]
- Howard et al. 2001 [32]
- Jho et al. 2002 [33]
- Kim et al. 2006 [34]
- Kim et al. 2013 [35]
- Krieghoff et al. 2006 [36]
- Lee et al. 2001 [9]
- Lewis 2003 [37]
- Mazemondet et al. 2011 [38]
- Meineke et al. 2001 [39]
- Monk 2003 [40]
- Orton et al. 2009 [41]
- Pralle et al. 2000 [42]
- Prior et al. 2003 [43]
- Rida et al. 2004 [44]
- Sakane et al. 2010 [45]
- Salic et al. 2000 [46]
- Swat et al. 2004 [47]
- Wawrzak et al. 2007 [48]
- Yamamoto et al. 2006 [49]
- Yamamoto et al. 2008 [50]

References

[1] Chen J, Xie ZR, Wu Y. Computational Modeling of the Interplay between Cadherin-Mediated Cell Adhesion and Wnt Signaling Pathway. PLoS ONE. 2014;9(6):e100702. doi:10.1371/journal.pone.0100702.

[2] Cho KH, Baek S, Sung MH. Wnt pathway mutations selected by optimal β-catenin signaling for tumorigenesis. FEBS Letters. 2006;580(15):3665–3670. doi:10.1016/j.febslet.2006.05.053.

[3] Goldbeter A, Pourquié O. Modeling the segmentation clock as a network of coupled oscillations in the Notch, Wnt and FGF signaling pathways. Journal of Theoretical Biology. 2008;252(3):574–585. doi:10.1016/j.jtbi.2008.01.006.

[4] Haack F, Lemcke H, Ewald R, Rharass T, Uhrmacher AM. Spatio-temporal Model of Endogenous ROS and Raft-Dependent WNT/Beta-Catenin Signaling Driving Cell Fate Commitment in Human Neural Progenitor Cells. PLoS Computational Biology. 2015;11(3):e1004106. doi:10.1371/journal.pcbi.1004106.

[5] Haack F, Budde K, Uhrmacher AM. Exploring the mechanistic and temporal regulation of LRP6 endocytosis in canonical WNT signaling. Journal of Cell Science. 2020;133(15). doi:10.1242/jcs.243675.

[6] Kim D, Rath O, Kolch W, Cho KH. A hidden oncogenic positive feedback loop caused by crosstalk between Wnt and ERK pathways. Oncogene. 2007;26(31):4571–4579. doi:10.1038/sj.onc.1210230.

[7] Kogan Y, Halevi-Tobias KE, Hochman G, Baczmanska AK, Leyns L, Agur Z. A new validated mathematical model of the Wnt signalling pathway predicts effective combinational therapy by sFRP and Dkk. Biochemical Journal. 2012;444(1):115–125. doi:10.1042/BJ20111887.

[8] Krüger R, Heinrich R. Model reduction and analysis of robustness for the Wnt/β-catenin signal transduction pathway. Genome Informatics. 2004;15(1):138–148. doi:10.11234/gi1990.15.138.

[9] Lee E, Salic A, Kirschner MW. Physiological regulation of β-catenin stability by Tcf3 and CK1ε. Journal of Cell Biology. 2001;154(5):983—-994. doi:10.1083/jcb.200102074.

[10] Mazemondet O, John M, Leye S, Rolfs A, Uhrmacher AM. Elucidating the Sources of β-Catenin Dynamics in Human Neural Progenitor Cells. PLoS ONE. 2012;7(8):e42792. doi:10.1371/journal.pone.0042792.

[11] Mirams GR, Byrne HM, King JR. A multiple timescale analysis of a mathematical model of the Wnt/β-catenin signalling pathway. Journal of Mathematical Biology. 2010;60(1):131–160. doi:10.1007/s00285-009-0262-y.

[12] Padala RR, Karnawat R, Viswanathan SB, Thakkar AV, Das AB. Cancerous perturbations within the ERK, PI3K/Akt, and Wnt/β-catenin signaling network constitutively activate inter-pathway positive feedback loops. Molecular BioSystems. 2017;13(5):830–840. doi:10.1039/C6MB00786D.

[13] Rodríguez-González J, Santillán M, Fowler A, Mackey MC. The segmentation clock in mice: interaction between the Wnt and Notch signalling pathways. Journal of theoretical biology. 2007;248(1):37–47. doi:10.1016/j.jtbi.2007.05.003.

[14] Sick S, Reinker S, Timmer J, Schlake T. WNT and DKK determine hair follicle spacing through a reaction-diffusion mechanism. Science. 2006;314(5804):1447–1450. doi:10.1126/science.1130088.

[15] Staehlke S, Haack F, Waldner AC, Koczan D, Moerke C, Mueller P, et al. ROS Dependent Wnt/β-Catenin Pathway and Its Regulation on Defined Micro-Pillars—A Combined In Vitro and In Silico Study. Cells. 2020;9(8):1784. doi:10.3390/cells9081784.

[16] van Leeuwen IMM, Byrne HM, Jensen OE, King JR. Elucidating the interactions between the adhesive and transcriptional functions of -catenin in normal and cancerous cells. Journal of Theoretical Biology. 2007;247(1):77–102. doi:10.1016/j.jtbi.2007.01.019.

[17] van Leeuwen IMM, Mirams GR, Walter A, Fletcher A, Murray P, Osborne J, et al. An integrative computational model for intestinal tissue renewal. Cell Proliferation. 2009;42(5):617–636. doi:10.1111/j.1365-2184.2009.00627.x.

[18] Wang Hy, Huang Yx, Qi Yf, Zhang Y, Bao Yl, Sun Lg, et al. Mathematical models for the Notch and Wnt signaling pathways and the crosstalk between them during somitogenesis. Theoretical Biology and Medical Modelling. 2013;10(1):27. doi:10.1186/1742-4682-10-27.

[19] Wawra C, Kühl M, Kestler HA. Extended analyses of the Wnt/β-catenin pathway: Robustness and oscillatory behaviour. FEBS Letters. 2007;581(21):4043–4048. doi:10.1016/j.febslet.2007.07.043.

[20] Bafico A, Liu G, Yaniv A, Gazit A, Aaronson SA. Novel mechanism of Wnt signalling inhibition mediated by Dickkopf-1 interaction with LRP6/Arrow. Nature Cell Biology. 2001;3(7):683–686. doi:10.1038/35083081.

[21] Bernard S, Čajavec B, Pujo-Menjouet L, Mackey MC, Herzel H. Modelling transcriptional feedback loops: the role of Gro/TLE1 in Hes1 oscillations. Philosophical Transactions of the Royal Society A: Mathematical, Physical and Engineering Sciences. 2006;364(1842):1155–1170. doi:10.1098/rsta.2006.1761.

[22] Bourhis E, Tam C, Franke Y, Bazan JF, Ernst J, Hwang J, et al. Reconstitution of a Frizzled8·Wnt3a·LRP6 Signaling Complex Reveals Multiple Wnt and Dkk1 Binding Sites on LRP6. Journal of Biological Chemistry. 2010;285(12):9172–9179. doi:10.1074/jbc.M109.092130.

[23] Brown KS, Hill CC, Calero GA, Myers CR, Lee KH, Sethna JP, et al. The statistical mechanics of complex signaling networks: nerve growth factor signaling. Physical Biology. 2004;1(3):184–195. doi:10.1088/1478-3967/1/3/006.

[24] Kwang-Hyun C, Sung-Young S, Hyun-Woo K, Wolkenhauer O, McFerran B, Kolch W. Mathematical Modeling of the Influence of RKIP on the ERK Signaling Pathway. In: Priami C, editor. Computational Methods in Systems Biology. Berlin, Heidelberg: Springer Berlin Heidelberg; 2003. p. 127–141.

[25] Dajani R, Fraser E, Roe SM, Yeo M, Good VM, Thompson V, et al. Structural basis for recruitment of glycogen synthase kinase 3beta to the axin-APC scaffold complex. The EMBO Journal. 2003;22(3):494–501. doi:10.1093/emboj/cdg068.

[26] Dequéant ML, Glynn E, Gaudenz K, Wahl M, Chen J, Mushegian A, et al. A Complex Oscillating Network of Signaling Genes Underlies the Mouse Segmentation Clock. Science. 2006;314(5805):1595—-1598. doi:10.1126/science.1133141.

[27] Galli LM, Barnes T, Cheng T, Acosta L, Anglade A, Willert K, et al. Differential inhibition of Wnt-3a by Sfrp-1, Sfrp-2, and Sfrp-3. Developmental Dynamics. 2006;235(3):681–690. doi:10.1002/dvdy.20681.

[28] Goh LK, Sorkin A. Endocytosis of Receptor Tyrosine Kinases. Cold Spring Harbor Perspectives in Biology. 2013;5(5):a017459–a017459. doi:10.1101/cshperspect.a017459.

[29] Hannoush RN. Kinetics of Wnt-driven β-catenin stabilization revealed by quantitative and temporal imaging. PLoS ONE. 2008;3(10). doi:10.1371/journal.pone.0003498.

[30] Hernández AR, Klein AM, Kirschner MW. Kinetic Responses of β-Catenin Specify the Sites of Wnt Control. Science. 2012;338(6112):1337–1340. doi:10.1126/science.1228734.

[31] Hirata H, Bessho Y, Kokubu H, Masamizu Y, Yamada S, Lewis J, et al. Instability of Hes7 protein is crucial for the somite segmentation clock. Nature Genetics. 2004;36(7):750—754. doi:10.1038/ng1372.

[32] Howard J, et al. Mechanics of motor proteins and the cytoskeleton. vol. 743. Sinauer associates Sunderland, MA; 2001.

[33] Jho Eh, Zhang T, Domon C, Joo CK, Freund JN, Costantini F. Wnt/β-Catenin/Tcf Signaling Induces the Transcription of Axin2, a Negative Regulator of the Signaling Pathway. Molecular and Cellular Biology. 2002;22(4):1172–1183. doi:10.1128/MCB.22.4.1172-1183.2002.

[34] Kim S, Xu X, Hecht A, Boyer TG. Mediator Is a Transducer of Wnt/β-Catenin Signaling*. Journal of Biological Chemistry. 2006;281(20):14066–14075. doi:10.1074/jbc.M602696200.

[35] Kim SE, Huang H, Zhao M, Zhang X, Zhang A, Semonov MV, et al. Wnt Stabilization of β-Catenin Reveals Principles for Morphogen Receptor-Scaffold Assemblies. Science. 2013;340(6134):867–870. doi:10.1126/science.1232389.

[36] Krieghoff E, Behrens J, Mayr B. Nucleo-cytoplasmic distribution of β-catenin is regulated by retention. Journal of Cell Science. 2006;119(7):1453–1463. doi:10.1242/jcs.02864.

[37] Lewis J. Autoinhibition with Transcriptional Delay: A Simple Mechanism for the Zebrafish Somitogenesis Oscillator. Current Biology. 2003;13(16):1398–1408. doi:10.1016/S0960-9822(03)00534-7.

[38] Mazemondet O, Hubner R, Frahm J, Koczan D, Bader B, Weiss D, et al. Quantitative and kinetic profile of Wnt/β-catenin signaling components during human neural progenitor cell differentiation. Cellular and Molecular Biology Letters. 2011;16(4):515—538. doi:10.2478/s11658-011-0021-0.

[39] Meineke FA, Potten CS, Loeffler M. Cell migration and organization in the intestinal crypt using a lattice-free model. Cell Proliferation. 2001;34(4):253–266. doi:10.1046/j.0960-7722.2001.00216.x.

[40] Monk NAM. Oscillatory Expression of Hes1, p53, and NF-κB Driven by Transcriptional Time Delays. Current Biology. 2003;13(16):1409–1413. doi:10.1016/S0960-9822(03)00494-9.

[41] Orton RJ, Adriaens ME, Gormand A, Sturm OE, Kolch W, Gilbert DR. Computational modelling of cancerous mutations in the EGFR/ERK signalling pathway. BMC Systems Biology. 2009;3(1):100. doi:10.1186/1752-0509-3-100.

[42] Pralle A, Keller P, Florin EL, Simons K, Hörber JKH. Sphingolipid–Cholesterol Rafts Diffuse as Small Entities in the Plasma Membrane of Mammalian Cells. Journal of Cell Biology. 2000;148(5):997—1008. doi:10.1083/jcb.148.5.997.

[43] Prior IA, Muncke C, Parton RG, Hancock JF. Journal of Cell Biology. 2003;160(2):165—-170. doi:10.1083/jcb.200209091.

[44] Rida PCG, Le Minh N, Jiang YJ. A Notch feeling of somite segmentation and beyond. Developmental Biology. 2004;265(1):2–22. doi:10.1016/j.ydbio.2003.07.003.

[45] Sakane H, Yamamoto H, Kikuchi A. LRP6 is internalized by Dkk1 to suppress its phosphorylation in the lipid raft and is recycled for reuse. Journal of Cell Science. 2010;123(3):360–368. doi:10.1242/jcs.058008.

[46] Salic A, Lee E, Mayer L, Kirschner MW. Control of Beta-Catenin Stability: Reconstitution of the Cytoplasmic Steps of the Wnt Pathway in Xenopus Egg Extracts. Molecular Cell. 2000; p. 10. doi:10.1016/S1097-2765(00)80446-3.

[47] Swat M, Kel A, Herzel H. Bifurcation analysis of the regulatory modules of the mammalian G1/S transition. Bioinformatics. 2004;20(10):1506–1511. doi:10.1093/bioinformatics/bth110.

[48] Wawrzak D, Métioui M, Willems E, Hendrickx M, de Genst E, Leyns L. Wnt3a binds to several sFRPs in the nanomolar range. Biochemical and Biophysical Research Communications. 2007;357(4):1119–1123. doi:10.1016/j.bbrc.2007.04.069.

[49] Yamamoto H, Komekado H, Kikuchi A. Caveolin Is Necessary for Wnt-3a-Dependent Internalization of LRP6 and Accumulation of β-Catenin. Developmental Cell. 2006;11(2):213–223. doi:10.1016/j.devcel.2006.07.003.

[50] Yamamoto H, Sakane H, Yamamoto H, Michiue T, Kikuchi A. Wnt3a and Dkk1 Regulate Distinct Internalization Pathways of LRP6 to Tune the Activation of β-Catenin Signaling. Developmental Cell. 2008;15(1):37–48. doi:10.1016/j.devcel.2008.04.015.
