# Supplementary figures and images for: Relating simulation studies by provenance—Developing a family of Wnt signaling models
Source: PLoS Comput Biol. 2021 Aug 5;17(8):e1009227. doi: 10.1371/journal.pcbi.1009227 (PMC8407594; doi:10.1371/journal.pcbi.1009227)

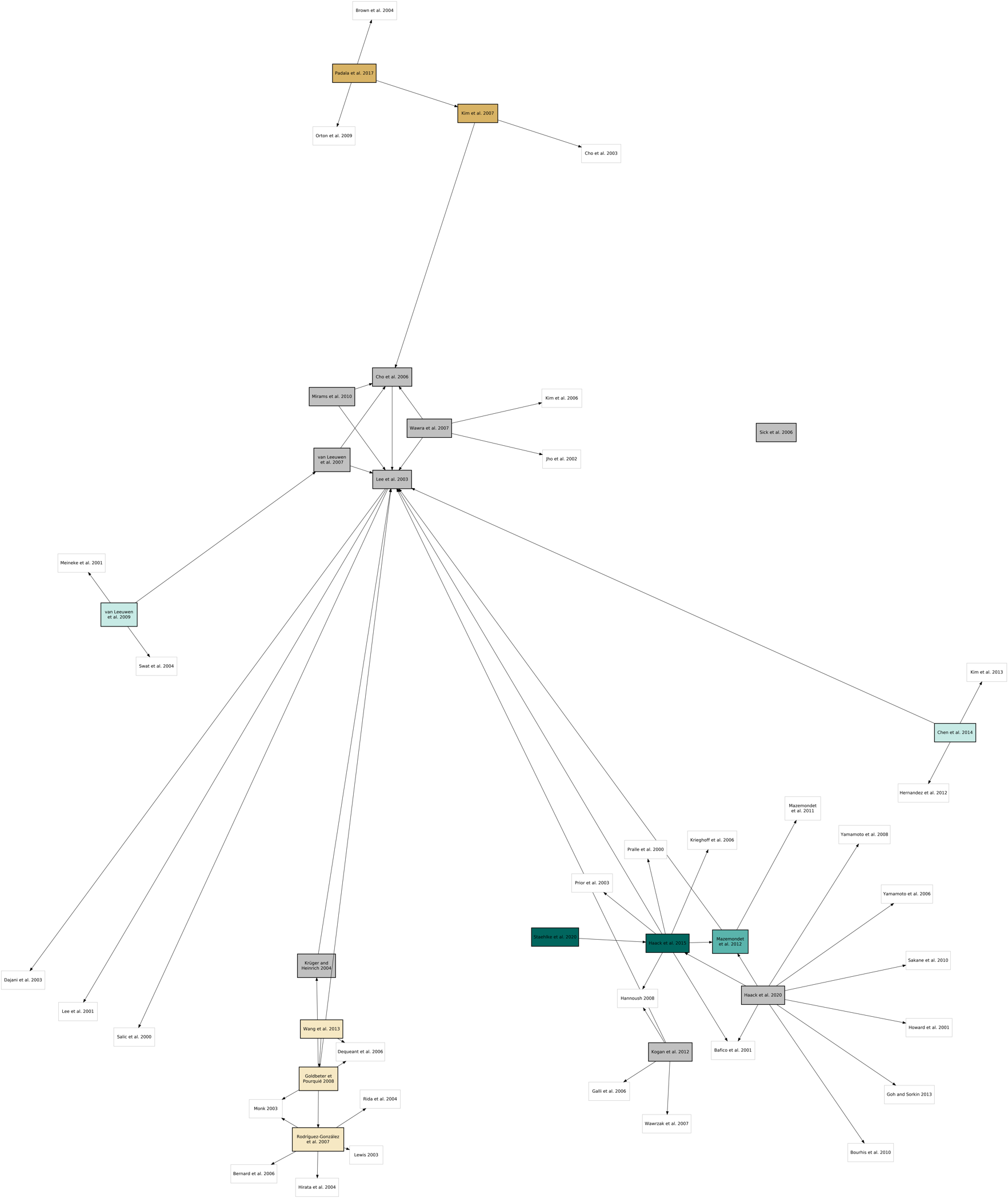

Supplement: S1 Fig — Studies which include additional pathways have been colored. (PDF) [file pcbi.1009227.s003.pdf]
