# Supplementary material for: Relating simulation studies by provenance—Developing a family of Wnt signaling models
Source: PLoS Comput Biol. 2021 Aug 5;17(8):e1009227. doi: 10.1371/journal.pcbi.1009227 (PMC8407594; doi:10.1371/journal.pcbi.1009227)
Supplement: Attachment — Submitted filename: response.pdf [file pcbi.1009227.s006.pdf]

Review response: “Relating simulation studies by provenance—Developing a family of Wnt signaling models”

Kai Budde, Jacob Smith, Pia Wilsdorf, Fiete Haack, Adelinde M. Uhrmacher

May 26, 2021

Dear Prof. Pedro Mendes,
Dear Prof. Jason Haugh,
Dear Prof. Chris J. Myers,
Dear anonymous reviewer,
Dear Dr. David P. Nickerson,

Thank you for your letter and for allowing us to revise our manuscript. We appreciate your thorough and constructive feedback! The questions and suggestions offered by the reviewers have been very helpful for improving our manuscript. We have split the reviews into separate points and discuss each of them. All page numbers refer to the revised manuscript file with tracked changes. We sincerely hope that we were able to address all concerns and incorporate necessary changes into our manuscript.

1st Reviewer

“ ”

Much has been made recently of the importance of reproducibility of scientific research. In biological simulation studies, such as this paper considers, this means providing the models and the analysis instructions in standard machine readable forms. This paper takes this a step further to look at the issue of provenance for these models. Namely, how are models related to previous models and experimental studies. In particular, the authors looks at a family of 19 models of the Wnt signaling pathway, which that manually link together using the PROV-DM ontology. To construct these relationships, they have developed a web tool, WebProv, to link studies with PROV-DM types and relations. As the authors point out, extracting these relationships from published studies is a highly laborious process that requires many assumptions along the way. Ideally, in the future, these provenance networks should be developed at the time of model construction during the simulation study.

This paper is an important demonstration of both the process of creating and utility of provenance networks for simulation studies. The prototype software tool presented should help facilitate future such activities. This proof-of-concept presented in this paper should become a tutorial for others that would undertaken this task for their models and simulation studies. The key issue remaining is how to motivate and facilitate others to create this information. This is not a problem that the authors can solve, but rather one that the community and the journal publishers should devote time and energy to in order to further improve the reproducibility of science.

Thank you, Prof. Myers. We agree with you and appreciate your kind feedback on the importance of our work.

2nd Reviewer

“ ”

This is an interesting paper that looks in detail at 19 Wnt related modeling papers. As a practicing

> modeler myself the most interesting pages started on page 14 (Provenance of individual Wnt simulation models) which discusses the various issues encountered, problems with the current ontolgies etc. I think this is the most important part of the paper from the point of view of the plos comp bio readership, such analyses have not been done as exhaustively as this one.
>
> Some of the material, particular the descriptions of the provenance entries (which seems to dominate the paper) could be summarized in a table and the textual component moved to an appendix. This would allow the reader to get straight to the most interesting papers of the paper. ...

Thank you, dear reviewer, for finding our work interesting! We agree with you that some readers might find the second part of the *Results and discussion* section more interesting than the first one. The first part of the section, entitled "Further steps towards a PROV-DM ontology for cellular biochemical simulation models", contains our definitions and examples of every entity and activity type used in our provenance data model. It belongs to the knowledge engineering part of our contribution, which is a prerequisite for the detailed provenance information of the Wnt models. We have extended the introductory paragraph of the *Results and discussion* section. This should make it clearer to the reader that the first part may be skipped by those who wish to jump directly to the Wnt model family. The changes are in lines 184–187.

> " "
>
> ... Caption: The captions to some of the figures could be improved.
>
> Fig 2: This caption starts with the word 'additionally' which doesn't sit well. Also the caption is too short, given that this is probably one of the more important figures. It took me a while to realize what the terms ASM, CSM etc meant (They were in Table 2). I would spell out these abbreviations (ASM, CSM, VSM, BSM) in the caption (Table 2 can remain unchanged), this will save the reader for having to search for their meaning. The caption would also add one sentence on how to read the figure. I know that earlier on the authors explain what an arrow means but that was some pages away and since plos comp bio are generally no computer scientists I would add that explanation of the arrow to the caption as well.
>
> Fig 3: In general, notation used in UML diagrams is not familiar to most modelers but in this case the diagram looks simple enough that its seems fairly self-explanatory. No action required. ...

We have adapted and extended the Fig 2 caption and added the abbreviations (BSM, QM, ...) to the legend of the figure (instead of the caption). We hope that this makes the content of the figure clearer to the reader.

> " "
>
> ... Minor: Typo in caption first sentence : 'prociding', I'm not sure what that word means, probably a typo but not sure what word should be there instead? ...

Thanks for pointing out the typo. We have fixed it.

> " "
>
> ... Software: As it stands it is likely that very few people will use WebProv, the reason is that it requires far too much work to install, plus does it also require a backend sever?
>
> The tool looks useful so why make it difficult to get hold of?
>
> What I would recommend is move everything if possible to the client (including the database which doesn't seem large) and host it as a github web page project so that when a user clicks on the url the application will show up, no installation necessary (which I think is one of the main attractions of web software) – see https://pages.github.com/. I strongly recommend something like this otherwise your work will not have the impact it should. ...

We were initially using GitHub Pages to deploy the first version of the software. However, we have decided to switch to Netlify since it offers better web hosting services. In addition to deploying the frontend to Netlify, the backend and database are now deployed on cloud servers which should make it easier for readers of the publication to get an impression of the tool and the provenance information of the Wnt models. Besides installing WebProv locally (see next paragraph), the tool can be accessed at `https://webprov.netlify.app`.

“ ”

. . . Documentation: There is no easily accessible documentation for the software. It looks like users are expected to download the github repo then select the indexl.html file in Docs. It would be better to host a proper (eg readthedocs) documentation on the github account itself. The only documentation link in the readme take a user to process manger 2 page. . . .

The documentation (usage and deployment) is located in the README on GitHub. We have modified it to make it more comprehensible. The `docs/` folder and `docs/index.html` file have been removed. This folder was previously used for GitHub Pages which deploys static webpages from the `docs/` folder.

“ ”

. . . Minor: 1. Introduction, second paragraph, first sentence, ‘conduction’, is that the right word? . . .

We have changed the wording (line 6).

“ ”

. . . 2. There is no reference to the youtube video on page 4 (footnote 2), also put the github ural there as well since the github repository is mentioned.

For most URLs, we have used hyperlinks. Thus, clicking on YouTube in the former footnote 2 should have led you to the website `https://youtu.be/UzwHtptkYOU`. We will make this video publicly available as soon as and only if the manuscript has been accepted for publication in PLOS Computational Biology.

As a side note: We have removed all footnotes and moved the information they contain to the main text as required by PLOS Computational Biology.

3rd Reviewer

“ ”

In this manuscript, the authors present a continuation of previous work to make use of the PROV-DM to represent provenance of biosimulation studies. The authors then use this data model to encode the provenance of a number of Wnt signalling models from the literature, and use the encoded provenance knowledge to examine the relationships between these published studies. The authors have also developed an open-source web-based tool providing a graphical user interface for creating, editing, exploring, and searching the provenance knowledge encoded in this manner. This seems to be a very useful approach for capturing provenance knowledge of systems biology modelling studies and appears to be extensible to capture richer provenance semantics as the collection/recording methods improve in future and this approach is applied in different domains or different types of models.

The authors should be commended for making all the software and data used in this manuscript freely available and documented in a manner sufficient to enable others to repeat the analysis presented here. . . .

Thank you, Dr. Nickerson! We are striving to publish all material related to our research and the publication.

“ ”

. . . I suggest the entire manuscript is thoroughly proof-read as some of the grammar and word choices are a bit unusual. ”cell biological systems” in the abstract is one example that could be tidied up. . . .

We have proofread the manuscript and corrected all mistakes. Minor changes can be found throughout the text. We hope that all language problems have been eliminated.

Additionally, we have slightly modified Fig 1. Instead of writing “Axin”, we now denote “Axin(2)” to show that both “Axin1” and “Axin2” are involved in Wnt signaling and are often used interchangeably in the simulation models. We have also clarified the link from sFRP to Wnt.

“ ”

... As the authors state, the knowledge they have extracted from the literature and encoded in the example provenance graph used in this work makes a useful contribution to the community of potential users of these Wnt signally models. I wonder if the authors have any plans or thoughts on the integration of this knowledge into a community repository, perhaps in a way that others could contribute to? For the subset of models that are available in the Biomodels database, for example, could the provenance knowledge be contributed back to the database? ...

As mentioned in our conclusion, we think that accessible provenance information would be a valuable extension of model repositories. Scharm et al. (2018) have also asked the maintainers of model repositories to add the possibility of entering provenance information: “We want to encourage the maintainers of repositories to provide a system where curators and modellers can transparently track the evolution of a project, e.g. using PROV-O to encode the provenance and COMODI to describe reason, intention, and effects of a change.” [1]

When comparing, for example, the entries of BioModels to our approach, the database contains provenance (meta)data of type Simulation Model and may additionally contain information of type Simulation Experiment and Simulation Data. The latter two usually comprise an experiment specification file, for instance a SED-ML file, as well as a figure showing the simulated data. In the case of the simulation models we have checked, the following BioModels entries contain information about the simulation experiments: the entry for Lee et al. (2003) [2] contains a SED-ML (and COPASI) file to reproduce parts of Fig 6 of that publication (corresponding to our SD6) and the entry for Padala et al. (2017) [3] contains the COPASI file to reproduce Fig 2A–C of that publication (corresponding to our SD3). The description in the overview section of a model entry might also include information about Research Questions and Assumptions. The other entities/activities and, more importantly, most relations are not available.

We are planning on contacting the BioModels team and would be happy to see (some of) the provenance data we have acquired to be added to the repository (see also next paragraph). However, all of this should be a community effort, which needs a broader discussion beforehand.

“ ”

... Following that thought, some of the provenance knowledge captured here is similar to that represented in the Biomodels database using the ”isDerivedFrom” predicate in the SBML model annotations (see for example the analysis of diabetes models presented in `https://dx.doi.org/10.1038%2Fpsp.2013.30`). Have the authors compared this knowledge for the subset of Wnt models available in the Biomodels database to see if similar (although less semantically rich) patterns of model evolution are present to their analysis presented in this manuscript? ...

Thank you for pointing out the publication by Ajmera et al. (2013). We have added this review to our paper in lines 32–34. A key finding of the review is the following: “The model relationship map (Figure 3) provides a complete overview of the evolution of most diabetes models available in the literature to-date and highlights the significance of sharing and reuse of models.” [4]

For some models presented in Figure 3 (of that publication), this relationship is also shown in BioModels using the qualifier *isDerivedFrom*: “The modelling object represented by the model element is derived from the modelling object represented by the referenced resource (modelling object B). This relation may be used, for instance, to express a refinement or adaptation in usage for a previously described modelling component.” [5]

When comparing this qualifier with our approach, we find that whenever we have a connection of the kind ‘Building Simulation Model (of simulation study i) $\longrightarrow$ Simulation Model (of simulation study j)’, we could add ‘model i *isDerivedFrom* model j’ to BioModels. An example is shown in Figure 1.

As we have written in our manuscript, “we have (...) not included the direct connection between two activities or two entities, such as the possibility to have a model being derived from another model. Thus, we have not included (...) `WasDerivedFrom`, which describes a direct transformation (update) of an entity into a new one.” However, the *isDerivedFrom* relation can be inferred from the information stored in the provenance graph.

Out of six Wnt models included in BioModels, two comprise information about model relations. First, the model by Kim et al. (2007) has *isDerivedFrom* information: ‘DOI 10.1007/3-540-36481-1_11’ (Cho et al.

2003) and ‘PubMed 1455190’ (Lee et al. (2003)). When comparing it with our provenance information, we have found an *isDerivedFrom*-equivalent connection to Cho et al. (2003) and Cho et al. (2006). The latter is missing in BioModels. Cho et al. (2006), on the other hand, is connected to Lee et al. (2003). (See our GitHub repository with additional files. This link has also been given in the main text.)

Second, the model by Padala et al. (2017) contains the following *isDerivedFrom* information: ‘BIOMD0000000623’ (Orton et al. (2009)), ‘BIOMD0000000033’ (Brown et al. (2004)), and ‘BIOMD0000000149’ (Kim et al. (2007)). All of these connections are also included in our provenance graph.

We will provide the BioModels team with further *isDerivedFrom* information that could be added to the annotations of the Wnt models in the repository. This information is extracted from our provenance graph by querying for a Building Simulation Model activity (of simulation study i) that *used* a Simulation Model entity (of another simulation study j).

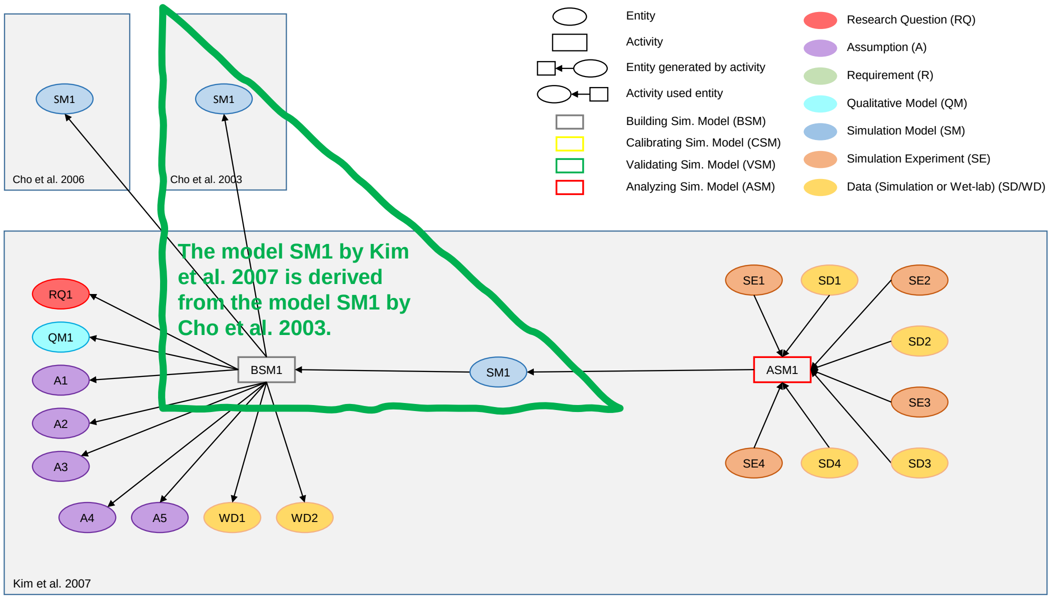

Figure 1: **Provenance graph of the study by Kim et al. (2007) [6].** Additionally, the three entities and their corresponding relations (SM1(Cho2003) ← BSM1(Kim2007) ← SM1(Kim2007)) are surrounded by a green triangle showing the possibility to extract *isDerivedFrom* information from provenance graphs.

“ ”

> … Using the SBO to annotate the assumptions seems an odd choice to me. Looking at Table S1, it seems that the SBO terms are giving a very high level annotation as to the type of model entity mentioned in the assumption, but doesn't provide any semantics about what the assumption is. Looking at assumptions annotated with SBO:0000009 (kinetic constant), for example, a user can search for assumptions that have something to do with a kinetic constant, but doesn't help to examine if its an assumption based on time scale analysis (e.g., row 3) or perhaps just an assumption that certain behaviour is assumed (e.g., row 13). I wonder if something like the Evidence and Conclusion Ontology (https://evidenceontology.org/) might provide a source of more meaningful terms to use in annotating assumptions? I may simply be missing something here, so perhaps a bit more explanation about how the SBO annotations are being used to annotate assumptions would help clarify things (or future work to extend the current work with enriched semantics?). …

You are right. Using SBO to annotate the (modeling) assumptions only shows which part of the model is being approximated (e.g., the kinetic constant, interactions of molecules, etc.). We have chosen SBO because it is "tailored specifically for the kinds of problems being faced in Systems Biology" [7]. We tried to clarify this in lines 240–241.

We have looked into the Evidence and Conclusion Ontology (ECO). It is "describing the various types of evidence that are generated during the course of a scientific study and which are typically used to support assertions made by researchers" [8]. When collecting the assumptions made by the authors, the evidence for these has not always become clear to us. Therefore, we do not feel able to annotate the assumptions using ECO.

“ ”

> … The authors define a minimal set of PROV-DM entities and activity types they have found useful for capturing provenance information of simulation studies when extracting provenance knowledge from the published literature. This minimal set does seem sufficient for the Wnt signalling demonstration presented here and the authors briefly explore how this set could be expanded in future. But I worry that the wet-lab data entity seems under-specified and perhaps less useful than it could be. While I understand that often in the literature the source of experimental data is not clearly described, with the recent growth of platforms like `https://www.protocols.io/` which enable scientists to provide rich descriptions of their protocols in a reusable manner, I wonder if the authors have considered how to incorporate that type of knowledge into their provenance graphs? …

We have decided not to include Wet-lab Experiments at this point, but only to consider the results of these experiments. In the future, one could add an entity of type Wet-lab Experiment as well as corresponding activities describing its generation. We know that information about the experiment itself is very important. Therefore, we added the possibility to point to the full wet-lab experiment description, for example, by referencing a file on `https://www.protocols.io/` or by providing a DOI within the description of a Wet-lab Data entity (see line 359).

“ ”

> … Minor comments
> --------------
> It may not be obvious to the reader exactly what PROV is when first mentioned in the abstract. …

We have changed it and hope that it is clearer now.

“ ”

... The assertion in the abstract that this provenance information is all that is required to answer the question of an ”appropriate starting point” is perhaps overstating things. The provenance information contributes to that answer, but it is not the only knowledge that is required to make an informed decision. ...

We have adapted our claim.

“ ”

... Figure 3 caption: ”prociding” - perhaps meant to be providing? ...

We have fixed this.

“ ”

... I completely agree with the authors that provenance information should be collected during the simulation study, but I wonder if the authors have given any thought to how their WebProv tool could be utilised as part of a typical modelling lifecycle to help encourage modellers to do so?

In our experience, it is best to capture provenance information (semi-)automatically or manually during the modeling (and simulation) process. We have added a paragraph discussing this matter (see lines 472–483).

References

[1] Scharm M, Gebhardt T, Touré V, Bagnacani A, Salehzadeh-Yazdi A, Wolkenhauer O, Waltemath D. Evolution of computational models in BioModels Database and the Physiome Model Repository. BMC Systems Biology. 2018;12(1):53. doi:10.1186/s12918-018-0553-2.

[2] Lee E, Salic A, Krüger R, Heinrich R, Kirschner MW. The Roles of APC and Axin Derived from Experimental and Theoretical Analysis of the Wnt Pathway. PLoS Biology. 2003;1(1):e10. doi:10.1371/journal.pbio.0000010.

[3] Padala RR, Karnawat R, Viswanathan SB, Thakkar AV, Das AB. Cancerous perturbations within the ERK, PI3K/Akt, and Wnt/β-catenin signaling network constitutively activate inter-pathway positive feedback loops. Molecular BioSystems. 2017;13(5):830–840. doi:10.1039/C6MB00786D.

[4] Ajmera I, Swat M, Laibe C, Le Novère N, Chelliah V. The impact of mathematical modeling on the understanding of diabetes and related complications. CPT: Pharmacometrics & Systems Pharmacology. 2013;2(7):54. doi:https://doi.org/10.1038/psp.2013.30.

[5] COMBINE consortium. isDerivedFrom (model qualifier);. Available from: `http://http://biomodels.net/model-qualifiers/isDerivedFrom`.

[6] Kim D, Rath O, Kolch W, Cho KH. A hidden oncogenic positive feedback loop caused by crosstalk between Wnt and ERK pathways. Oncogene. 2007;26(31):4571–4579. doi:10.1038/sj.onc.1210230.

[7] Namespace: Systems Biology Ontology; 2021. Available from: `https://registry.identifiers.org/registry/sbo`.

[8] Chibucos MC, Siegele DA, Hu JC, Giglio M. In: The Evidence and Conclusion Ontology (ECO): Supporting GO Annotations. New York, NY: Springer New York; 2017. p. 245–259.
